# Inflammation and Microbiota Regulation Potentiate Pneumonia Therapy by Biomimetic Bacteria and Macrophage Membrane Nanosystem

**DOI:** 10.34133/research.0096

**Published:** 2023-03-27

**Authors:** Yuan Li, Xiangmei Liu, Zhenduo Cui, Yufeng Zheng, Hui Jiang, Yu Zhang, Zhaoyang Li, Shengli Zhu, Paul K Chu, Shuilin Wu

**Affiliations:** ^1^ School of Materials Science and Engineering, Peking University, Beijing 100871, China.; ^2^ The Key Laboratory of Advanced Ceramics and Machining Technology by the Ministry of Education of China, School of Materials Science and Engineering, Tianjin University, Tianjin 300072, China.; ^3^ Biomedical Materials Engineering Research Center, Hubei Key Laboratory of Polymer Materials, Ministry-of-Education Key Laboratory for the Green Preparation and Application of Functional Materials, School of Materials Science and Engineering, Hubei University, Wuhan, 430062, China.; ^4^ School of Health Science and Biomedical Engineering, Hebei University of Technology, Xiping Avenue 5340, Beichen District, Tianjin 300401, China.; ^5^ Department of Orthopedics, Guangdong Provincial People’s Hospital, Guangdong Academy of Medical Sciences, Guangzhou, 510080, China.; ^6^ Department of Physics, Department of Materials Science and Engineering, and Department of Biomedical Engineering, City University of Hong Kong, Kowloon 999077, Hong Kong, China.

## Abstract

While conventional nanosystems can target infected lung tissue, they cannot achieve precise cellular targeting and enhanced therapy by modulating inflammation and microbiota for effective therapy. Here, we designed a nucleus-targeted nanosystem with adenosine triphosphate (ATP) and reactive oxygen species stimuli–response to treat pneumonia coinfected with bacteria and virus that is enhanced through inflammation and microbiota regulation. The nucleus-targeted biomimetic nanosystem was prepared through the combined bacteria–macrophage membrane and loaded hypericin and ATP-responsive dibenzyl oxalate (MMHP) subsequently. The MMHP despoiled the Mg^2+^ of intracellular cytoplasm in bacteria to achieve an effective bactericidal performance. Meanwhile, MMHP can target the cell nucleus and inhibit the H1N1 virus duplication by inhibiting the activity of nucleoprotein. MMHP possessed an immunomodulatory ability to reduce the inflammatory response and activate CD8^+^ T cells for assisted infection elimination. During the mice model, the MMHP effectively treated pneumonia coinfected with *Staphylococcus aureus* and H1N1 virus. Meanwhile, MMHP mediated the composition of gut microbiota to enhance the pneumonia therapy. Therefore, the dual stimuli-responsive MMHP possessed promising clinical translational potential to therapy infectious pneumonia.

## Introduction

Recently, influenza A virus (H1N1) and bacteria coinfections in pneumonia have become a global health problem because they have induced more than 95% of severe illnesses and death [1,2]. The influenza infection was complicated and deadly with secondary bacterial infection [1–5]. The most common means of treatment nowadays was combined drug therapy [6–11]. However, traditional drug therapy by taking antivirus and antibacterial antibiotics can simultaneously cause multiple side effects and much uncertain drug conflict [12–15]. Compared with influenza A- or bacteria-caused pneumonia alone, pneumonia coinfected with influenza A virus and bacteria was fickle and can lead to higher lethality [16,17]. They were able to cause greater immune system damage to the infected organism, which also limited the effects of the drug [18–20]. Therefore, more effective and safe therapy means were urgently exploited to treat pneumonia that is coinfected with virus and bacteria. Although a number of drug-carrying nanosystems have been developed, developing precise nanosystems to improve drug transport into infected tissues was still a key challenge.

There are also other nanomaterials such as gel nanoparticles, mesoporous silicon (MN), or carbon spheres used in reactive oxygen species (ROS)-responsive nanomaterials for acute lung injury or pneumonia [21,22]. Although they also enable intelligent drug delivery and clearance of infection, they have some drawbacks in terms of biocompatibility and efficiency of release of the loaded drug [22]. Biomimetic nanocarriers were exploited for drug delivery for the past few years [23–29]. Among them, the macrophage membrane was more used for drug loading because of its hydrophilia, safety, and drug loading capacity [30–32]. The receptors on the membrane surface also gave the function of cell membrane-coated NPs for immunoregulation similar to a real macrophage [33,34]. When the NPs were loaded by the macrophage membrane, the NPs can be transported through the blood and then released under an acidic and infected environment [35,36]. Moreover, the bacterial outer membrane was also a drug delivery nanosystem well-separated from bacillus strains [37,38]. The outer membrane vesicles inherited partial membrane protein and can be used as antigens in consequence [39,40]. They can also activate immunoreaction of infected patients in vivo except for loaded drug [30,31,41].

**Fig. 1.**
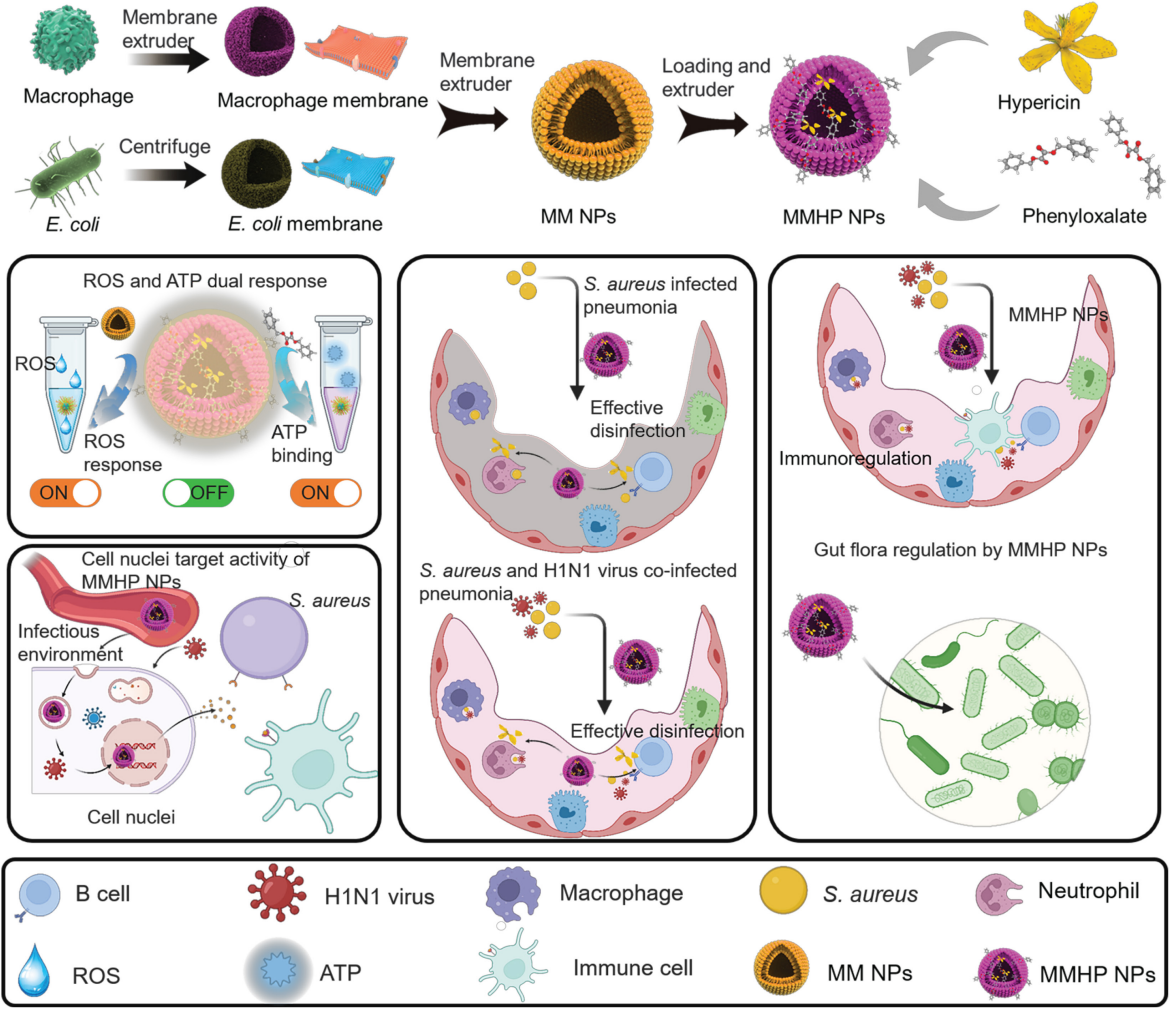
The mechanism of precise pneumonia therapy with MMHP NPs. The nucleus-targeted biomimetic nanosystem (MMHP) was prepared through the combined bacteria–macrophage membrane and loaded ATP-responsive molecular (dibenzyl oxalate) and hypericin (HP). The loaded HP despoiled the Mg^2+^ of intracellular cytoplasm in bacteria to achieve an effective bactericidal effect. MMHP NPs targeted the cell nucleus and inhibited the H1N1 virus duplication by inhibiting the activity of nucleoprotein. Benefited from natural immunoregulation of HP and immunogenicity of the combined bacteria–macrophage membrane, MMHP NPs possessed an immunomodulatory ability to reduce the inflammatory response and recruit CD8^+^ T cells for assisted infection elimination. Meanwhile, MMHP NPs mediated the composition of gut microbiota to amplify the pneumonia therapy.

Considering the similar composition structure and different efficacy of bacteria membrane and macrophage membrane [36,42], we designed a hybrid drug-carrying membrane structure compounded by them (Fig. 1). The dual stimuli-responsive nanosystem (consisting of RAW 264.7 membrane and *Escherichia coli* membrane) was loaded with ROS- and adenosine triphosphate (ATP)-responsive molecular (dibenzyl oxalate) and hypericin (HP) with a nucleus-targeting ability. The loaded HP was controlled to be released by changing the external stimulation including ROS or ATP concentration. Through the broad-spectrum antimicrobial performance, we found that the bacteria–macrophage membrane and loaded hypericin and ATP-responsive dibenzyl oxalate (MMHP) NPs despoiled the Mg^2+^ from bacteria to realize an effective bactericidal effect. When the HP interacted with H1N1 virus-infected cells, they targeted the nuclei and inhibited the H1N1 virus duplication by inhibiting the activity of nucleoprotein (NP). The mechanism of MMHP NPs treating H1N1- and *Staphylococcus aureus*-infected pneumonia was shown in Fig. 1. Because of its responsive capability toward ATP and ROS, the MMHP NPs can release loaded HP effectively with target activity. Therefore, abundant HP was released under the infected lung position. On one hand, the MMHP eradicated infected *S. aureus* and H1N1 effectively because of its antibacterial, antiviral, and immunomodulatory properties. On the other hand, MMHP modulated cell-mediated immunity and the composition of the gut microbiota. Therefore, the designed dual-response MMHP NPs had a better ability to treat infectious diseases and related clinical application prospects.

## Results

### ROS- and ATP-responsive activity of MMHP NPs

**Fig. 2.**
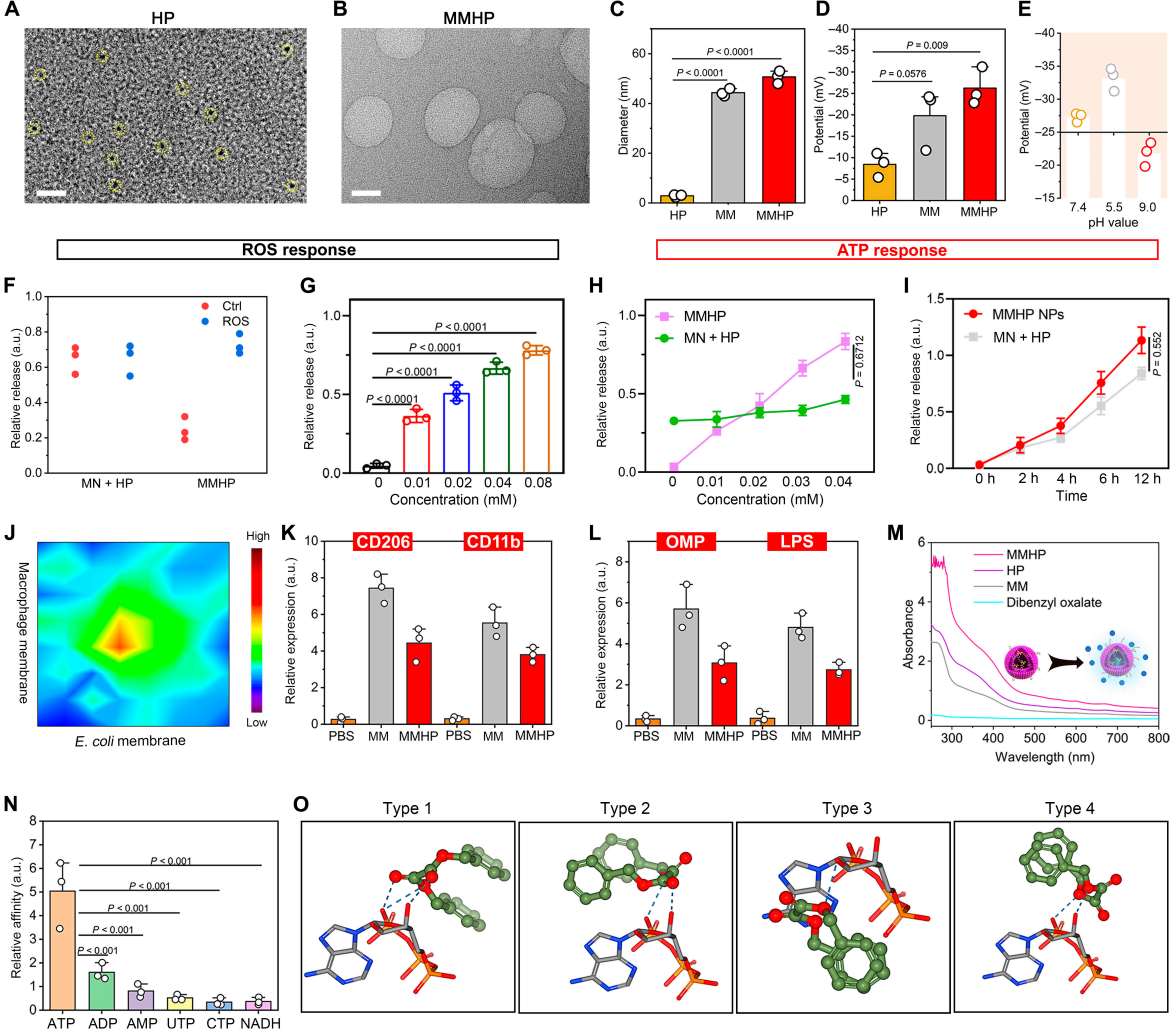
The ROS- and ATP-responsive activity of MMHP NPs. (A) The TEM image of HP. Scale bar, 20 nm. (B) The TEM image of MMHP NPs. Scale bar, 20 nm. (C) The dynamic light scattering size of MM, HP, and MMHP NPs. (D) The zeta potential of MM, HP, and MMHP NPs. (E) The varied zeta potential of MMHP NPs at pH 7.4, 5.5, and 9.0, respectively. (F) The release of HP of MN + HP and MMHP under neutral (Ctrl) or ROS (H_2_O_2_) environment. (G) The release of HP in under varied ROS (H_2_O_2_) environment from 0.01 to 0.08 mM. (H) The release of HP under different ATP concentrations from 0.01 to 0.04 mM. MN + HP means the physical mixture of MN (mesoporous silicon) and HP. (I) The release of HP under varied time. (J) The release of HP under varied *E. coli* and macrophage membrane ratio. (K) The CD206 and CD11b protein expressions of PBS, MM, and MMHP NPs. (L) The LPS and OMP protein expression of PBS, MM, and MMHP NPs. (M) The ultraviolet-visible spectra of HP, MM, dibenzyl oxalate, and MMHP NPs from 300 to 800 nm. (N) Varied HP release of MMHPs under ATP and its decomposition product environment. (O) The interaction between ATP and dibenzyl oxalate. (C), (D), (G) to (I), (K), and (L) were analyzed with one-way ANOVA. a.u., arbitrary units.

First, the mixed membrane (MM) including the macrophage membrane and the bacterial membrane was extracted through an extruder and centrifugation process, respectively. Afterward, HP was loaded inside MM through an extruder for 40 cycles. ATP-responsive dibenzyl oxalate was loaded further to form MMHP NPs for more accurate drug delivery. On the basis of the transmission electron microscope (TEM) (JEM-2100F) images, we found that the HP and MMHP NPs possessed uniform sizes (Fig. 2A and B and Fig. S1A). The dynamic light scattering size of MMHP NPs was detected and showed a similar size (about 53.1 nm) with the observed size in the TEM image (Fig. 2C). The zeta potential of MM was negative, about −19.5 mV, and MMHP NPs became smaller, about −26.4 mV, indicating the electronegativity of HP (Fig. 2D). Under acidic (pH = 5.5) and alkaline (pH = 9.0) environment, the zeta potential became smaller and larger compared with the neutral environment (pH = 7.4), respectively (Fig. 2E). This was because of the increased permeability of the MM under an acidic environment, which led to the release of more HP and dibenzyl oxalate. They were partially adsorbed on the surface of the MMHP NPs, resulting in a decrease in zeta potential. On the contrary, as fewer particles were released under an alkaline environment, a larger zeta potential was presented. Then, we measured the ROS responsiveness of MMHP NPs by comparing the HP release amount under the neutral (Ctrl) condition and the ROS (H_2_O_2_) condition [43]. We found that the release of HP and MM was constant under neutral and ROS environments for HP, but MMHP was released more under the ROS environment, which indicated stellar responsiveness of MMHP to ROS (Fig. 2F). Compared with the MN + HP group (physical mixture of MN and HP), the released HP amount of MMHP NPs was increased with the increase of H_2_O_2_ concentration (0 to 0.08 mmol l^−1^) because of the enhanced permeability of MMHP NPs (Fig. 2G). However, because most of the HP in MN + HP lacked the response of ROS, it can be released in large amounts under the Ctrl and ROS environments. We examined the relative release of HP under different pH conditions, which included a slightly acidic environment (pH = 5.5), a weakly slightly acidic environment (pH = 6.5), and a neutral environment (pH = 7.4). According to the results, it can be seen that, under inflammatory conditions, the release of HP gradually increased with decreasing pH, which also indicates the responsive release ability of MMHP under inflammatory conditions (Fig. S1B). Then, we examined the release of HP as the culture time gradually increased from 1 to 3 d. According to the results, it can be seen that under inflammatory conditions, the release of HP gradually increased with the increased incubation time, which also indicates the responsive release ability of MMHP under inflammatory conditions (Fig. S1C). Afterward, the ATP-responsive activity of MMHP NPs was also evaluated. Similarly, the release of HP in MMHP NPs was obviously enhanced with gradient ATP concentrations (0 to 0.04 mmol l^−1^). With increased incubation time, the released HP of MMHP NPs was enhanced and higher than the MN + HP group invariably under an ATP environment (ATP concentration = 0.02 mmol l^−1^) (Fig. 2H and I). Then, we changed the proportion of RAW 264.7 cell membrane and *E. coli* membrane to investigate the best drug delivery efficiency of the MM nanosystem (Fig. 2J). When the proportion of the RAW264.7 membrane and the *E. coli* membrane reached 1:1, we found the highest release of HP. The drug loading rate of the hybrid cell membrane drug-loading carriers is related to the stability of the overall structure and size. Because bacterial membranes and macrophage membranes are of different origins, because of their size and structural inconsistency, when their ratio is higher than 1:1 or lower than 1:1, the difference in their contents leads to stress differences in the process of extrusion and fusion to form a hybrid cell membrane, resulting in a decrease in the loading rate compared to 1:1. The receptor of the MM in MMNPs was then detected (Fig. 2K and L). After the HP was coated by MM, the expression of CD206 and CD11b of MMHP NPs was slightly lower than RAW 264.7 but higher than PBS. Similarly, the expression of outer membrane protein (OMP) and lipopolysaccharide (LPS) in MMHP NPs was lower than that in MM but higher than that in phosphate-buffered saline (PBS). It indicated that the membranes of RAW 264.7 and *E. coli* were successfully coated. The ultraviolet-visible (UV-2700, Shimadzu) spectrum was used to verify the successful loading of HP (Fig. 2M). We can see that MM and HP had a broad but weak absorption peak between 300 and 400 nm, but the absorption of MMHP was enhanced compared with both, indicating that MMHP was the composite of MM and HP. Although it was not possible to determine the successful loading of dibenzyl oxalate, it can be derived from the previous ATP-responsive release. Compared with the decomposition product of ATP (including adenosine diphosphate, adenosine monophosphate, uridine triphosphate, cytidine triphosphate, and nicotinamide adenine dinucleotide), ATP possessed the highest binding ratio with dibenzyl oxalate, indicating that MMHP NPs had response properties under the ATP environment (Fig. 2N). To understand the potential principle, we performed a theoretical calculation based on density functional theory. Through simulating the interaction of dibenzyl oxalate and ATP, we found that there was 4 different combination modes between them (Fig. 2O and Table S1). Because of the formed strong binding force of the hydrogen bond, the PO_4_^3−^ of ATP was combined with the molecular that induced HP release. Therefore, when MMHP NPs were under the environment of ATP, ATP was able to compete with dibenzyl oxalate molecules in the structure of MMHP NP, thus leading to its structural disruption and causing massive HP release. Then, we evaluated the antioxidant activity of MMHP using radical scavenging activities. According to the experimental results of 2,2-diphenyl-1-picrylhydrazyl radical scavenging, it can be found that NPs have a weak radical scavenging ability, but the radical scavenging efficiency of MMHP NPs was greatly enhanced at 10 min after being loaded by MM, which also indicates the excellent antioxidant ability of the prepared MMHP NPs (Fig. S1D).

### The antibacterial activity and corresponding mechanism of MMHP NPs

**Fig. 3.**
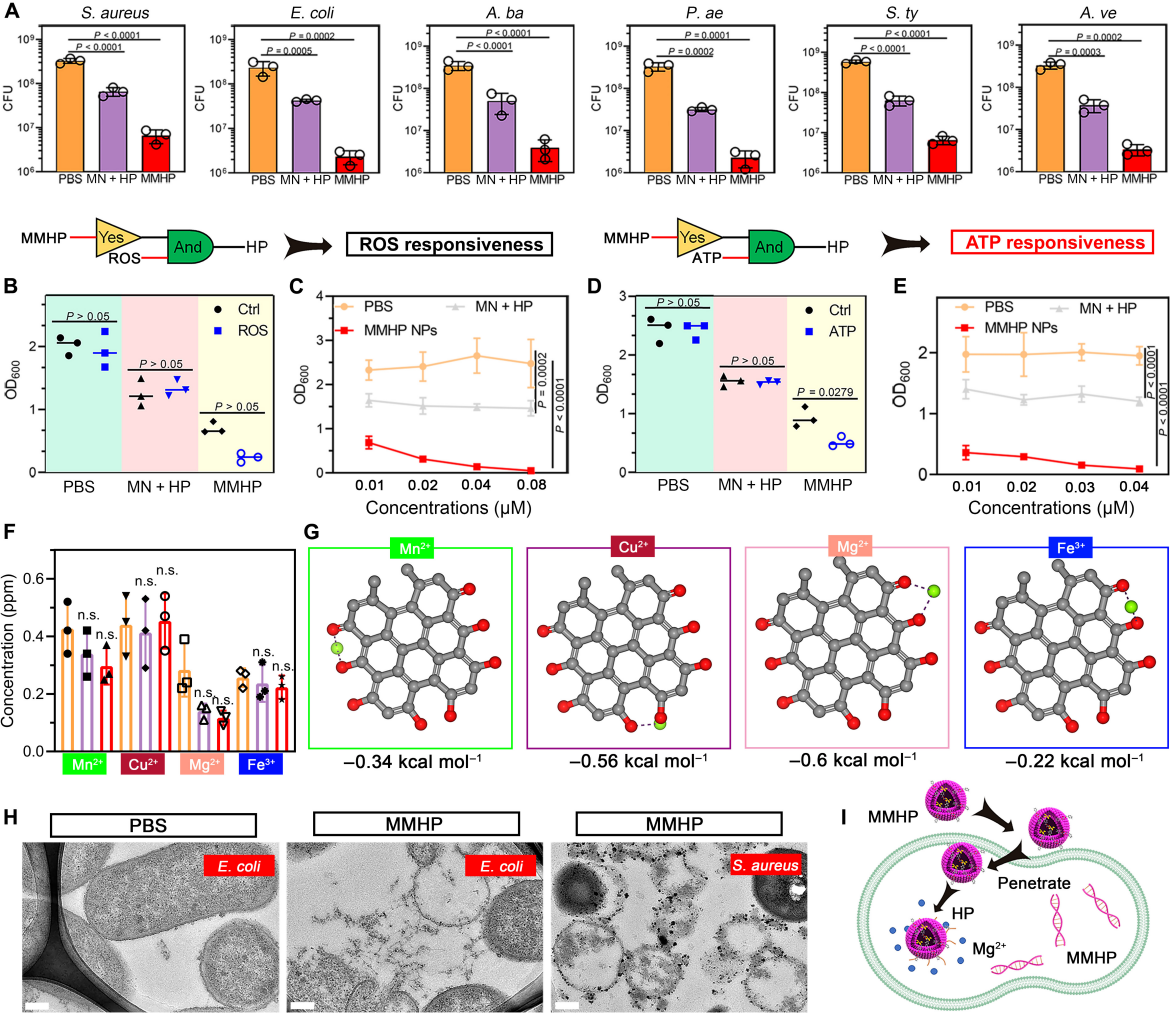
The antibacterial activity and corresponding mechanism of MMHP NPs toward broad-spectrum bacteria. (A) The antibacterial activity of PBS, MN + HP, and MMHP NPs toward different bacterial strains. (B) The optical density (600 nm) of PBS, MN + HP, and MMHP NPs under neutral or ROS (H_2_O_2_) environment. (C) Varied optical densities (600 nm) of PBS, MN + HP, and MMHP NPs treated by PBS, MN + HP, and MMHP NPs at different ROS concentrations (0.01 to 0.08 mM). (D) The optical density (600 nm) of PBS, MN + HP, and MMHP NPs under varied ATP concentrations. (E) The optical density (600 nm) of PBS, MN + HP, and MMHP NPs during varied ROS concentrations (0.01 to 0.04 mM). (F) The Mn^2+^, Cu^2+^, Mg^2+^ and Fe^3+^ concentrations of PBS-, HP-, and MMHP NP-treated *S. aureus*. (G) The molecular docking between Mg^2+^ and HP. (H) The TEM image of *S. aureus* and *E. coli* after PBS and MMHP NP treatments. (I) The antibacterial mechanism of MMHP NPs. (A) to (F) were analyzed with one-way ANOVA. n.s., not significant.

Then, we evaluated the broad-spectrum antibacterial efficiency toward strains including *S. aureus*, *E. coli*, *Acinetobacter baumannii* (*A. ba*), *Pseudomonas aeruginosa* (*P. ae*), *Salmonella typhimurium* (*S. ty*), and *Aeromonas veronii* (*A. ve*). First, we found that HP had a well antibacterial effect (97.9% toward *S. aureus* and 98.2% toward *E. coli*), but MM had almost no antibacterial effect (Fig. S2). Therefore, we measured the antimicrobial properties of the designed MMHP nanosystem under dual response by comparing the antimicrobial effects of MN + HP and MMHP NPs. The MN + HP means the physical mixture of MN and HP. After a 24-h incubation of PBS, MN + HP, and MMHP NPs and bacterial solution, the colony-forming unit (CFU) was decreased from 10^8^ to 10^6^ CFU ml^−1^ toward MMHP NPs, which achieved the highest inhibition ratio (98.1% toward *S. aureus*, 99.1% toward *E. coli*, 98.8% toward *A. ba*, 99.4% toward *P. ae*, 99.3% toward *S. ty*, and 99.1% toward *A. ve*) (Fig. 3A). In contrast, the antimicrobial efficiency of MN + HP (79.9% toward *S. aureus*, 81.8% toward *E. coli*, 85.5% toward *A. ba*, 90.5% toward *P. ae*, 89.0% toward *S. ty*, and 88.5% toward *A. ve*) was lower than that of MMHP NPs probably because of the massive release of MMHP resulting from the dual response under ROS and ATP. However, the release of the MN + HP group was lower. We also observed the scanning electron microscope morphology of the bacteria after interacting with PBS and MMHPs (Fig. S3). The surface showed a complete shape in the PBS group and was plicated and damaged in MMHP NPs group.

Then, we investigated the antibacterial activity of MMHP NPs under varied ROS and ATP concentrations (Fig. 3B to E). We found that only MMHP NPs showed superior antimicrobial performance under the ROS environment. The antimicrobial performance for the PBS and MN + HP groups under neutral and ROS environments remained almost the same (Fig. 3B). Moreover, MMHP NPs exhibited increased antimicrobial properties as the concentration of ROS increased and distinguished with the PBS and MN + HP groups (Fig. 3C). Meanwhile, the MMHP NPs exhibited a similar responsive activity toward ATP (Fig. 3D and E). With the addition of exogenous ATP, the MMHP NPs presented better antibacterial activity at higher concentrations of ATP. As a result, the MMHP NPs possessed well-responsive ROS and ATP antibacterial activity. Furthermore, the antibacterial mechanism was also investigated. Different kinds of metal ions including Mn^2+^, Cu^2+^, Mg^2+^, and Fe^3+^ in *S. aureus* were detected after incubating with PBS, MN + HP, and MMHP NPs; we found an obvious decrease in Mg^2+^ content (Fig. 3F). Because Mg^2+^ was a necessary element during the bacterial metabolism process and protein synthesis, the homeostasis of Mg^2+^ was vital in live bacteria [44–46]. The affected Mg^2+^ metabolism can lead to disordered bacterial metabolism. It indicated that the MMHP NPs despoil the Mg^2+^ from the bacteria, and we verified that with molecular dynamics. The molecular docking between metal ions and HP was then carried out to prove the mechanism we raised (Fig. 3G). To compare with the binding force between them, the Mg^2+^–HP compound presented the lowest force of about −0.6 kcal mol^−1^ (Table S2). It indicated that Mg^2+^ was easier combined with HP in the bacterial cytoplasm. To observe the membrane morphology after interacting with MMHP NPs, we collected the TEM images of *E. coli* and *S. aureus* after the antibacterial process with MMHP NPs (Fig. 3H and Fig. S4A). A clear damaged membrane was shown during MMHP NP-treated *E. coli* and *S. aureus*. However, the membrane morphology showed to be rounded and intact in PBS-treated *E. coli* and *S. aureus*. The corresponding mechanism diagram was shown in Fig. 3I. The released HP interacted with bacteria and permeated into the bacteria inside. Because of the high affinity of HP for Mg^2+^, the inner HP despoiled the Mg^2+^ and affected the normal metabolic process of bacteria.

### The cell nuclei-targeted capability of MMHP NPs and corresponding antivirus mechanism

**Fig. 4.**
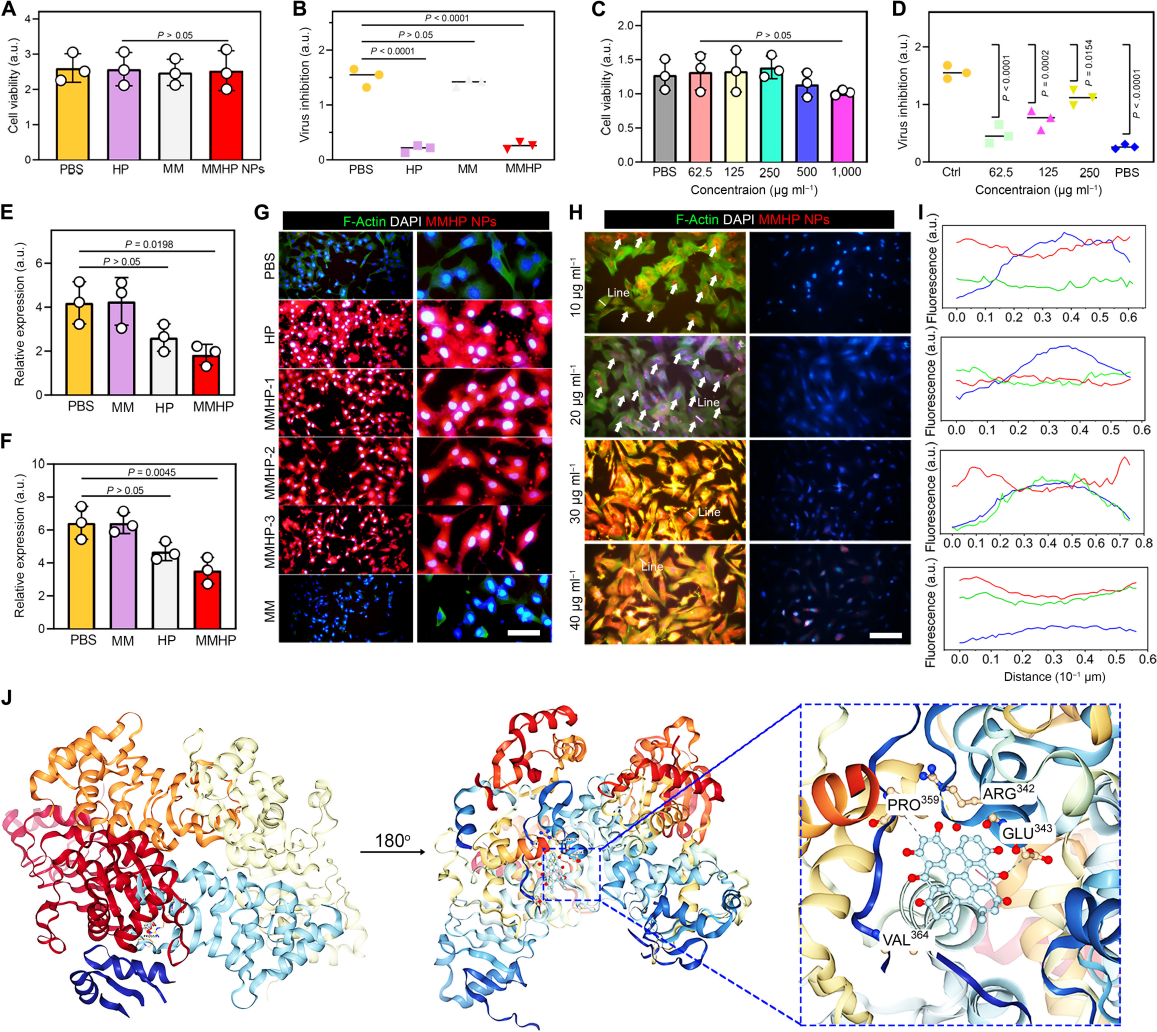
The cell nuclei-targeted capability of MMHP NPs and corresponding antivirus mechanism. (A) The MTT assay of PBS, HP, MM, and MMHP NPs toward A549 cells after 1 d of incubation. (B) The antivirus assay of PBS, HP, MM, and MMHP NPs toward A549 cells with H1N1 virus after 1 d of incubation. (C) The MTT assay of MMHP NPs with different concentrations toward A549 cells after 1 d of incubation. (D) The antivirus assay of MMHP NPs with different concentrations toward A549 cells with H1N1 virus after 1 d of incubation. The Ctrl group means that the cells were incubated without H1N1 virus. (E) The ELISA of NP protein expressions of PBS, HP, MM, and MMHP NPs. (F) The NP gene expressions of PBS, HP, MM, and MMHP NPs. (G) The fluorescence staining images of A549 cells (the concentrations of HP, MMHP-1, MMHP-2, MMHP-3, and MM were 250, 125, 250, 500, and 250 μg ml^−1^, respectively). Scale bar, 25 μm. (H) The fluorescence staining images of A549 cells with lower MMHP NP concentration. Scale bar, 20 μm. (I) The fluorescence intensity of green, blue, and red fluorescence along with the lines. (J) The molecular docking between NP and HP. (A) to (F) were analyzed with one-way ANOVA.

In consideration of the antivirus activity of HP and MMHP NPs, we evaluated the inhibition capability against the common H1N1 virus clinically. First, we evaluated biocompatibility and surveyed the appropriate concentration of MMHP NPs to inhibit the H1N1 virus. As shown in Fig. 4A, HP, MM, and MMHP (100 μg ml^−1^) presented similar methyl thiazolyl tetrazolium (MTT) results compared with the PBS group, indicating the bioactivity of MMHP NPs. We then measured the antiviral performance of MMHP NPs by coculturing MMHP NPs with A549 cells infected with the H1N1 virus [47]. We found that HP and MMHP NPs possessed 87.8% and 84.5% antivirus activity, and the efficiency of the MM alone was negligible (Fig. 4B). However, when the concentration of MMHP was increased to 500 μg ml^−1^, it presented 13.1% (500 μg ml^−1^) and 22.4% (1000 μg ml^−1^) cell cytotoxicity (Fig. 4C). Then, we investigated the antivirus activity of MMHP NPs under different concentrations (62.5, 125, and 250 μg ml^−1^). With the enhancement of MMHP NP concentration, the inhibition capability toward the H1N1 virus was also enhanced correspondingly (Fig. 4D). The highest antivirus activity reached 84.2% at 250 μg ml^−1^. The nucleoprotein (NP) expressions of the virus that represented the ability of the virus to replicate were furtherly detected to investigate the corresponding mechanism of MMHP NPs [48–50]. As shown in Fig. 4E, MMHP NPs reduced the NP protein expression compared with PBS and MM. Similarly, the NP gene detected by quantitative reverse transcription polymerase chain reaction (Bio-Rad) was also decreased in the MMHP NPs group (Fig. 4F). To get an insight into the penetrative activity of MMHP NPs, the cells were stained with blue fluorescence (represented nuclei) and green fluorescence (represented actin). Moreover, the red color represented the spontaneous fluorescence of MMHP NPs (Fig. 4G and H). On the basis of the spreading forms of filopodia and lamellipodia, the cells were not affected by MM, HP, and MMHP NPs (Fig. 4G). Under a low concentration of MMHP NPs, we found that the NPs first appeared near the A549 nuclei (Fig. 4H). Under a higher concentration of MMHP NPs, the cells were presented with full red fluorescence. On the basis of the fluorescence images (Olympus) of MMHP and A549 cells coincubated, the cell uptake efficiency of MMHP NPs was calculated on the basis of fluorescence intensity. After the incubation of MMHP NPs and A549 cells infected by the H1N1 virus, MMHP NPs showed higher fluorescence intensity compared with the HP and MM group; this was because more MMHP NPs were uptaken by A549 cells. Because of the ability to target the nucleus of HP, MMHP and HP can be rapidly internalized upon contact with cells (Fig. S4B).

The corresponding quantitative fluorescence intensity indicated that MMHP NPs can target the cell nuclei well (Fig. 4I). This demonstrated the ability of the MMHP to target the nucleus. Because the infected virus was usually the first to transfect and replicate at the nucleus site, MMHP NPs were well-positioned to rapidly inhibit virus replication and kill the virus. Furthermore, we verified the interaction of HP and NP protein in the H1N1 virus through molecular docking (Fig. 4J and Table S3). Moreover, obvious hydrogen bond interaction was shown between HP and Pro^359^, Arg^342^, Glu^343^, and Val^364^ ligands.

### MMHP NPs showed immunoregulation in pneumonia mice model

**Fig. 5.**
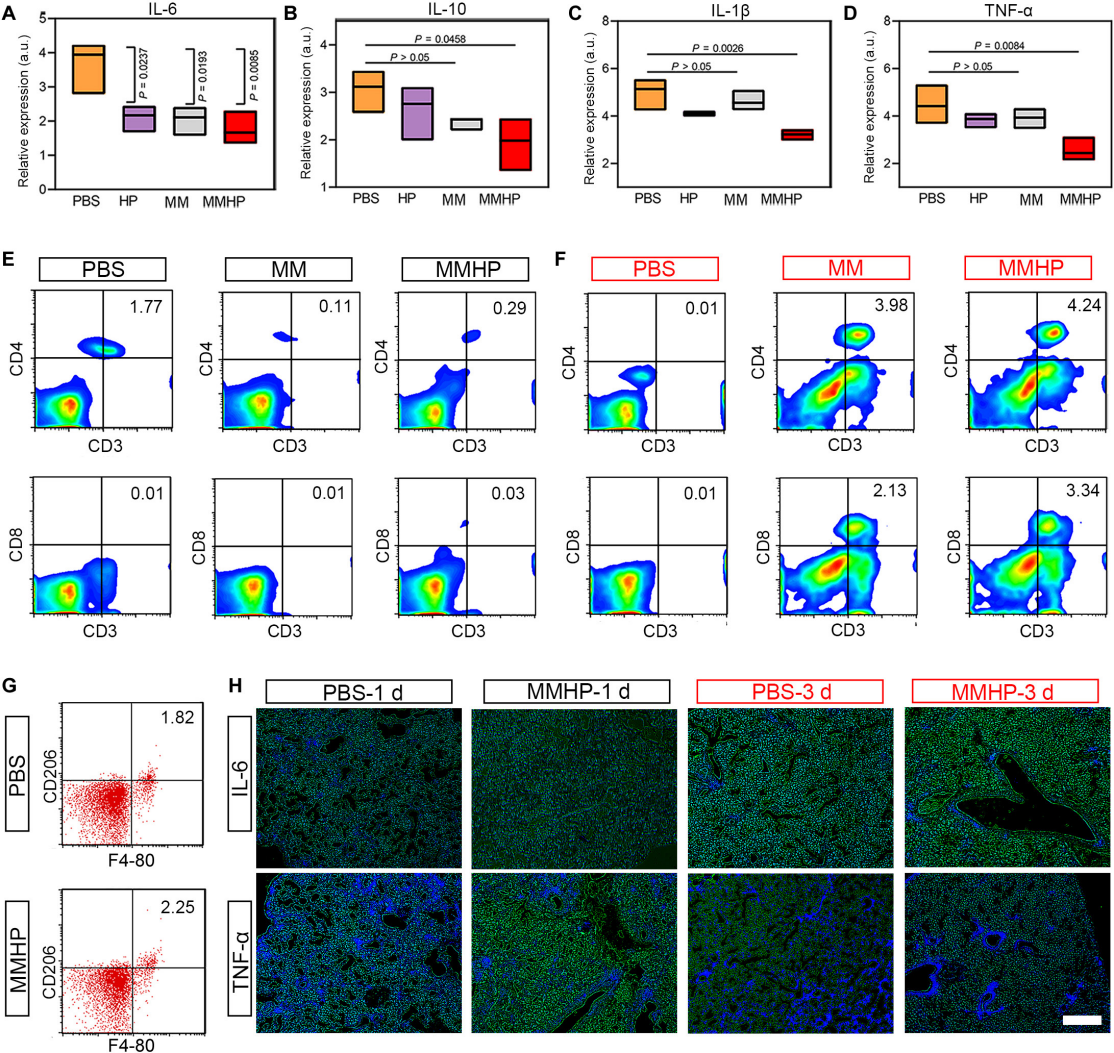
MMHP NPs showed immunoregulation in pneumonia mice model. (A to D) The inflammatory factor expressions of IL-6, IL-10, IL-1β, and TNF-α. (E) The CD4/CD8 flow cytometry of PBS-, MM-, and MMHP NP-treated lung tissues after 1 d of *S. aureus* infection. (F) The CD4/CD8 flow cytometry of PBS-, MM-, and MMHP NP-treated lung tissues after 3 d of *S. aureus* infection. (G) The CD206/F4-80 flow cytometry of PBS-, MM-, and MMHP NP-treated lung tissues after *S. aureus* infection at 3 d. (H) The IL-6 and TNF-α fluorescence staining after 1 and 3 d of infection. The green fluorescence means IL-6 or TNF-α. Scale bar, 20 μm. (A) to (D) were analyzed with one-way ANOVA.

Immunoregulation was another vital capability of therapeutic NPs [51,52]. In response to this, mice pneumonia models infected with *S. aureus* were established to evaluate the immunoregulation capability of MMHP NPs. First, the safety of MMHP NPs was detected through hematoxylin and eosin (H&E) staining of the main organs (Fig. S5). On the basis of the results of H&E staining, it can be seen that MMHP NPs did not show significant physiological toxicity in mice, which indicated the safety of MMHP NPs and the feasibility of a clinical application. After 1 d of infection, the PBS, MM, and MMHP NPs were inhaled through the nose using a nebulizer for 30 min. The inflammatory factors and blood routine examination in serum were first analyzed to evaluate the anti-inflammation activity (Fig. 5A to D and Fig. S6). Obviously, decreased expressions of interleukin-6 (IL-6), IL-1β, and tumor necrosis factor–α (TNF-α) can be observed in MM- and MMHP NP-treated mice. Similar results can be seen in blood routine tests. Therefore, we can tentatively determine that MMHP NPs had an immunomodulatory ability. Then, we went on to analyze their specific mechanism affecting immune regulation by flow cytometry (BD). We investigated the CD3/CD4/CD8 triple-marked cells in serum after 1 and 3 d of treatment (Fig. 5E and F). At the initial 1 d, MM and MMHP NPs mediated the CD8^+^ T cells (the double-positive expression of CD4/CD8) little compared with the PBS group (Fig. 5E). After 3 d, a clear increase of CD8^+^ cells was shown, indicating an immunoregulation activity in vivo (Fig. 5F). Because CD8^+^ T cells had immune memory and the ability to kill infected pathogens, MMHP NPs were highly effective to recruit immune cells to assist to kill infected bacteria and virus effectively in vivo. Then, we also investigated the CD206/F4-80 expressions that represented the macrophage cells in serum (Fig. 5G). We found that MMHP NPs increased about 25.6% of macrophage compared with the PBS group. The corresponding immunofluorescent staining of IL-6 and TNF-α, which was mainly expressed by macrophages, also proved it (Fig. 5H). Therefore, MMHP NPs mediated well the immunoreaction in vivo through up-regulated CD8^+^ T and macrophage cells. The antioxidant properties of MMHP NPs are explained by the inflammatory factor expressions of IL-6, IL-10, IL-1β, and TNF-α and immunofluorescence staining in vivo. The antioxidant property of MMHP NPs was produced by the combined bacteria–macrophage membrane and loaded HP [53]. On one hand, the surface of the combined bacteria–macrophage membrane is rich in a large number of receptors and signaling molecules, and the composite cell membrane not only adsorbs endotoxins but also accepts inflammatory cytokines as signaling molecules. The membranes are able to suppress the immune response and finally are able eliminate ROS by inhibiting the expression of major histocompatibility complex 2 after inflammatory activation. On the other hand, the loaded chrysin was able to suppress the inflammatory response by inhibiting the expression that suppresses oligomeric Aβ42 upon release from the inflammatory environment, thus significantly reducing the expression levels of IL-1β, IL-6, and TNF α [54].

### The therapeutic efficacy of MMHP NPs against primary H1N1 and *S. aureus* as well as coinfected pneumonia

**Fig. 6.**
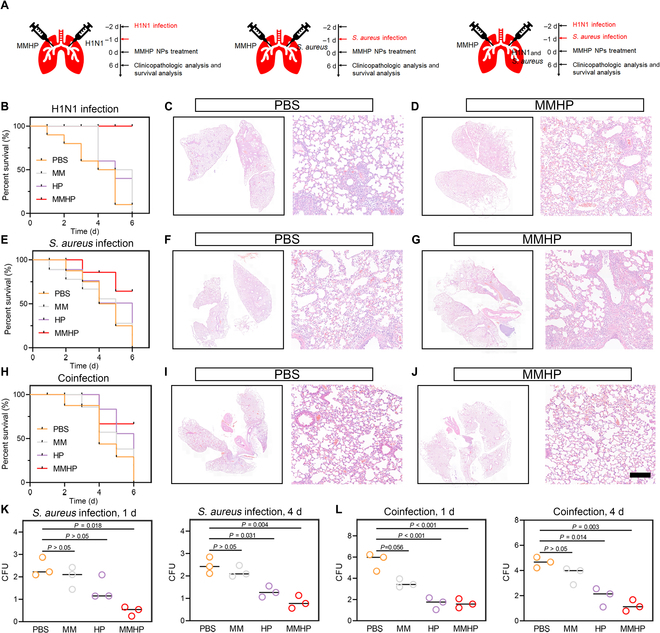
The therapeutic efficacy of MMHP NPs against primary H1N1 and *S. aureus* as well as coinfected pneumonia. (A) The mechanism of administration schedule toward 3 kinds of lung infection and treatment. (B) Survivorship curve of PBS-, MM-, HP-, and MMHP NP-treated mice with H1N1 infection. (C and D) H&E staining of lung tissue after PBS and MMHP NP therapy. (E) Survivorship curve of PBS-, MM-, HP-, and MMHP NP-treated mice with *S. aureus* infection. (F and G) H&E staining of lung tissue after PBS and MMHP NP therapy. (H) Survivorship curve of PBS-, MM-, HP-, and MMHP NP-treated mice with mixed H1N1 and *S. aureus* infection. (I and J) H&E staining of lung tissue after PBS and MMHP NP therapy. Scale bar, 20 μm. (K) The CFU in lung tissue of *S. aureus*-infected mice after 1 and 4 d. (L) The CFU in lung tissue of mixed H1N1 and *S. aureus*-infected mice after 1 and 4 d. (K) and (L) were analyzed with one-way ANOVA.

To further evaluate the antimicrobial and the antiviral capacity of MMHP NPs, single H1N1 or *S. aureus* infected mice with pneumonia, and pneumonia mice models coinfected with H1N1 virus and *S. aureus* were established. The corresponding experimental design diagram was shown in Fig. 6A; the mice were first infected by the H1N1 virus or *S. aureus* and were treated with MMHP NPs or PBS. Subsequently, the mice were sacrificed and analyzed through survival mice, pathology, and corresponding colony counting. During the 3 different infection types, MMHP NPs achieved higher survival in mice and lower lung inflammation in H&E staining images (100% survival in H1N1 virus infection, 64.3% survival in *S. aureus* infection, and 66.7% survival in coinfection) (Fig. 6B to J). On the basis of the results of H&E pathology sections, we were able to find a significant inflammatory phenomenon in the PBS-treated mice, with the presence of a large number of inflammatory cells. However, in the lungs of MMHP-treated mice, there was only a small amount of inflammation present, which was mainly caused by the accumulation of MMHP in the lung tissue and the antibacterial results. It can be seen that MMHP has a significant therapeutic effect on different types of pneumonia infections. To further verify the antibacterial activity of MMHP NPs in vivo, we counted the CFU in the lung tissue of PBS-, MM-, HP-, and MMHP NP-treated mice. After 1 d of treatment, about 76.8% of *S. aureus* were eradicated by MMHP NPs compared to the untreated PBS group (Fig. 6K). After 4 d of treatment, we found that negligible *S. aureus* can be found in MMHP NPs (Fig. 6L). However, about 10^5^ CFU ml^−1^ was grown in the PBS-treated mouse lung. Moreover, on the basis of the H&E sections of the lungs and trachea at 5 d, we can find that the mice in the MMHP NPs group were free of inflammation (Fig. S7). As a result, MMHP NPs showed therapeutic efficacy against single H1N1 or *S. aureus* and coinfected pneumonia.

### MMHP NPs mediated gut microbiota

**Fig. 7.**
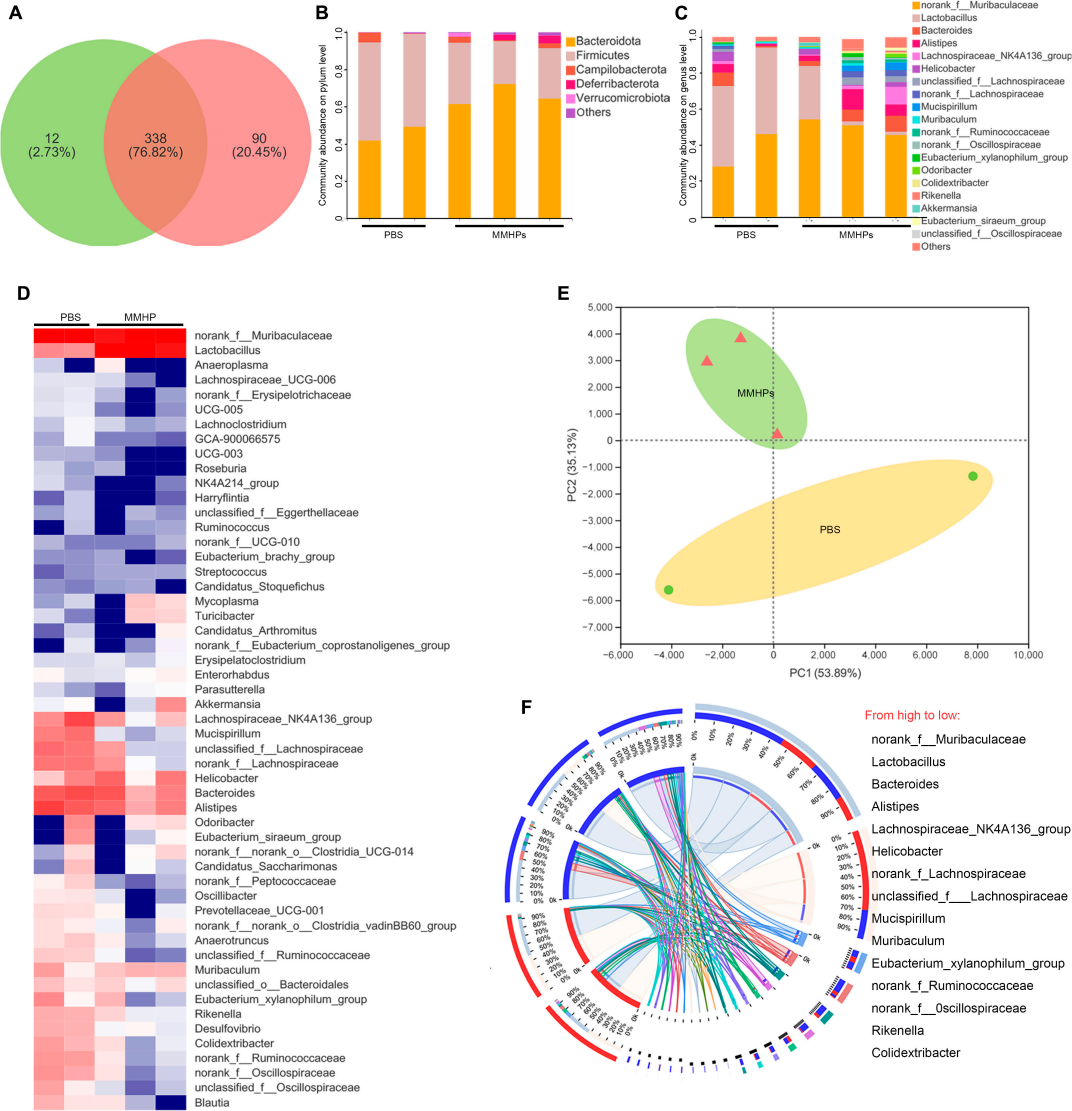
MMHP NPs mediated gut microbiota. (A) The Venn diagram shows the number of bacterial strains in infected mice’ intestinal tract. (B) The principal components analysis plot showing the distinct separation based on the bacterial strains’ profiles of PBS and MMHP NPs. (C) Differential bacterial strain heat maps of PBS- and MMHP NP-treated mice. (D) The differential bacterial strains of PBS- and MMHP NP-treated mice. (E) The composition of differential bacterial strains of PBS- and MMHP NP-treated mice. (F) The cladogram of gut microbiota treated by PBS and MMHP NPs.

Considering that gut microbiota played an important role during the process of maintaining normal pulmonary homeostasis, we carried out 16S ribosomal RNA sequencing to study the variation of diversity and composition caused by MMHP NPs. The Venn graph depicted 2 groups between PBS and MMHP NPs of overlapping OTU (operational taxonomic unit) data (Fig. 7A). Ninety bacterial strains were regulated after MMHP NP therapy. We found that the PBS group and the MMHP NPs group possessed unique gut microbiota structures at genus and phylum levels (Fig. 7B and C). Specifically, the caused diversity and composition were shown in the heat map (Fig. 7D). MMHP NPs increased Bacteroidota but decreased Firmicutes. Moreover, the principal coordinate analysis showed a relative similarity of diverse gut microbiota composition (Fig. 7E). The linear discriminant research was further carried out to analyze the changes between PBS- and MMHP-treated mice. We found that Bacteroidota was enriched during the MMHP NP-treated group but not in the PBS-treated group, based on the alpha diversity of gut microbiota about sobs, Shannon, simpson, ace, chao, coverage, and qstat. The values of sobs were similar to ace and chao, indicating that MMHP NPs did not affect the microbial diversity of mice (Table S4).

## Discussion

The conventional clinical treatment against infection is antibiotics, but they have the disadvantages of lack of intelligent drug delivery and side effects such as the destruction of intestinal flora. Here, we designed a nucleus-targeted biomimetic nanosystem (compounded by bacteria membrane and macrophage membrane) with ATP and ROS responsiveness for accurate pneumonia therapy. HP loaded in MMHP NPs was effectively released responsively with good linearity under the stimulation of ROS and ATP. To verify the dual responsiveness of this bionanocarrier, we analyzed the amount of HP released by varying the concentration of ROS and ATP to demonstrate its intelligent responsiveness. Then, we found that the fluorescence intensity of the material around the nucleus increased with the increase of the concentration of the material during coculture with cells, which indicates its nucleus-targeting property, which is crucial to enhance its antiviral ability. In addition, the bionanocarrier can fully exert its antibacterial and antiviral properties in the in vivo treatment of bacterial and viral coinfection in a mouse pneumonia model. At the same time, its anti-inflammatory properties are also beneficial for rapid pathogen clearance. The dual stimuli-responsive bacteria and macrophage membrane-coated NPs with a nucleus-targeted ability possessed well clinical translation capabilities to therapy complicated pneumonia.

Developing precise nanocarriers to improve drug transport into infected tissues and to the final site of action remains a key challenge. The conventional clinical treatment against infection is antibiotics, but they have the disadvantages of lack of intelligent drug delivery and side effects such as the destruction of intestinal flora. The biomimetic nanodrug delivery system, modified by the mixed bacteria–macrophage cell membrane, inherited the advantages of NPs and the receptors on the natural cell membrane surface, which can effectively avoid recognition and clearance by the immune system, thus prolonging the in vivo circulation time of the used drug. Considering the responsive nature of the inflammatory environment of MMHP NPs, the MMHP NPs can be used in the therapy of inflammatory diseases and infectious diseases, including enteritis, pneumonia, and infected wounds. MMHPs are able to utilize their self-microbial properties and modulate the inflammatory microenvironment of patients to synergistically and rapidly promote the clearance of infections. At the same time, by modifying the membrane of mixed bacteria–macrophage cells with additional loading of ROS-responsive molecules, such as loading sodium bicarbonate buffers on the surface, it is possible to achieve a lower release under noninflammatory environments while enhancing release under inflammatory ones. Here, we designed a nucleus-targeted biomimetic nanosystem (compounded by bacteria membrane and macrophage membrane) with ATP and ROS responsiveness for accurate pneumonia and enteritis therapy. HP loaded in MMHP NPs was effectively released responsively with good linearity under the stimulation of ROS and ATP. After the HP was coated by MM, the expression of CD206 and CD11b of MMHP NPs was slightly lower than RAW 264.7 but higher than PBS. Similarly, the expression of OMP and LPS in MMHP NPs was lower than that in MM but higher than that in PBS. It indicated that the membrane of RAW 264.7 and *E. coli* were successfully coated. Meanwhile, the zeta potential became smaller and larger compared with neutral environment (pH = 7.4) because of the increased permeability of the MM under an acidic environment, which led to the release of more HP and dibenzyl oxalate. They were partially adsorbed on the surface of the MMHP NPs, resulting in a decrease in zeta potential. On the contrary, as fewer particles were released under an alkaline environment, larger zeta potential was presented.

With the addition of exogenous ATP, the MMHP NPs presented better antibacterial activity at higher concentrations of ATP. As a result, the MMHP NPs possessed well-responsive ROS and ATP antibacterial activity. The released HP interacted with bacteria and permeated into the bacteria inside. Because of the high affinity of HP for Mg^2+^, the inner HP despoiled the Mg^2+^ and affected the normal metabolic process of bacteria. When the MMHP NPs interacted with cells infected with the H1N1 virus, they targeted the nuclei first and inhibited the H1N1 virus duplication by inhibiting the activity of the NP protein. Moreover, obvious hydrogen bond interaction was shown between HP and Pro^359^, Arg^342^, Glu^343^, and Val^364^ ligands. Moreover, the designed MMHP NPs possessed better immunoregulatory ability through recruiting CD8^+^ T cells and macrophages compared with MM alone. In the mice model, the MMHP NPs rescued *S. aureus*- and H1N1-infected pneumonia mice because of the antibacterial, antiviral, and immunomodulatory properties of MMHP NPs. The dual stimuli-responsive bacteria and macrophage membrane-coated NPs with a nucleus-targeted ability possessed well clinical translation capabilities to therapy complicated pneumonia.

In summary, we designed a dual stimuli-responsive nanosystem (consisting of RAW 264.7 membrane and *E. coli* membrane), which was loaded with ROS- and ATP-responsive molecular (dibenzyl oxalate) and HP with a nucleus-targeting ability. The loaded HP was controlled to be released by changing the external stimulation including ROS or ATP concentration. The MMHP NPs despoiled the Mg^2+^ from bacteria to realize an effective bactericidal effect. When the HP interacted with H1N1 virus-infected cells, they targeted the nuclei and inhibited the H1N1 virus duplication by inhibiting the activity of the nucleoprotein (NP). The MMHP effectively eradicated infected *S. aureus* and H1N1 because of its antibacterial, antiviral, and immunomodulatory properties. Meanwhile, they modulated cell-mediated immunity activity and the composition of gut microbiota. Therefore, the designed dual-response MMHP NPs had a better ability to treat infectious diseases and related clinical application prospects.

## Materials and Methods

### Preparation of MMHP NPs

First, RAW 264.7 cell membranes were extracted with an extruder for 20 cycles and saved at 4 °C for use. Moreover, the bacterial membrane of *E. coli* (at 10^9^ CFU ml^−1^) was extracted after high-speed centrifugation at 12,000 rpm. Afterward, HP of about 100 μg ml^−1^ was loaded in an MM solution to get membrane-coated HP NPs. Then, stirring was carried out at room temperature, and the stirring speed was kept at 500 rpm. After 12 h of stirring, the centrifugation was carried out at 10,000 rpm and 10 min with 3 washes of water and finally dried under vacuum at 50 °C. Then, the dibenzyl oxalate of 40 μg ml^−1^ (50 ml) was added to the membrane-coated HP NPs (2 mg ml^−1^, 100 ml) loaded on the surface of the NPs after stirring for 2 h to get MMHP NPs. Then, stirring was carried out at room temperature and the stirring speed was kept at 500 rpm. After 12 h of stirring, the centrifugation was carried out at 10,000 rpm and 10 min with 3 washes of water and finally dried under vacuum at 50 °C.

### Responsive activity of ROS and ATP

Both MMHP and MM were first configured into a solution with a concentration of 200 μg ml^−1^. With different ROS concentrations (H_2_O_2_ concentration from 0 to 0.08 mM), the released HP was detected with a microplate reader (BioTek, Synergy H1) after incubation of MMHP and H_2_O_2_. The standard curve is plotted by different concentrations of HP, and thus, the amount of HP released is calculated from the optical density (OD) value detected by the enzyme marker. In addition, we changed the ATP concentration (concentration from 0 to 0.04 mM) in the MMHP NP solution (200 μg ml^−1^), and the released HP was detected with the microplate reader. The incubation was changed from 0 to 12 h. The standard curve was also plotted by different concentrations of HP, and thus, the amount of HP released is calculated from the OD value detected using the microplate reader.

### Antibacterial activity of MMHP NPs

The MMHP NPs were configured to a concentration of 200 μg ml^−1^ and stored at 4 °C. Different bacterial strains (*S. aureus*, ATCC 25923; *E. coli*, ATCC 25922; *A. ba*, AB 2018157; *P. ae*, ATCC 15692; *S. ty*, CCTCC PB 2019001; and *A. ve*, ATCC 35624) were incubated at 37 °C to 10^9^ CFU ml^−1^ and then diluted to 10^5^ CFU ml^−1^. Afterward, the MMHP NPs and MN + HP (physical mixture of MM and HP) groups were coincubated with the bacterial strains for the next 24 h, and the OD values were detected finally. Concerning the ROS and ATP responsive activity, the ROS and ATP concentration was changed from 0.01 to 0.08 mM. Then, the OD value was also detected to evaluate the logic gate modulation.

### Inductively coupled plasma detection

The MMHP NPs were configured to a concentration of 200 μg ml^−1^ and stored at 4 °C. After the antibacterial process, the bacterial solution was centrifuged and nitrated to get the mixture. Then, the MMHP NPs were coincubated with the *S. aureus* strains for the next 24 h. The different Zn^2+^, Fe^3+^, Mn^2+^, and Mg^2+^ concentrations in *S. aureus* after MMHP treatment were detected with inductively coupled plasma mass spectrometry (iCAP RQ).

### Cell viability assay

The MMHP NPs were configured to a concentration of 200 μg ml^−1^ and stored at 4 °C. The samples were cocultured with A549 cells for 1 d. Then, 200 μl of methyl thiazolyl tetrazolium solution (the concentration was 2.5 mg ml^−1^) was added to the 96-well plate where the MMHP was cocultured with A549 cells and incubated at 37 °C for another 4 h. Then, the methyl thiazolyl tetrazolium solution was discarded and 200 μl of dimethyl sulfoxide solution was added and shaken for next 15 min. Finally, the OD of 100 μl of supernatant was measured on a microplate reader (at 490 nm). The cellular activity of the samples can be calculated on the basis of the ratio of OD values. For cell fluorescence assay, the samples including MM, HP, and MMHP, and incubated A549 cells were rinsed with PBS and fixed with 4% formaldehyde for 10 min at room temperature. The supernatant was discarded and was stained with fluorescein isothiocyanate dye (Solarbio, Beijing) for 30 min in the dark. Then, the solution was discarded and rinsed 3 times with PBS, followed by 4′,6-diamidino-2-phenylindole dye (Solarbio, Beijing) for 30 s, and then rinsed 3 times with PBS. After 2 h, the cell fluorescence images of the MM, HP, and MMHP groups were taken by inverted fluorescence microscopy (Olympus Corporation, Japan).

### Antivirus activity detection

The antivirus activity was detected by evaluating cell viability. The A549 cells were first infected by the H1N1 virus for 1 h. Then, the cell medium was changed and incubated with MM, HP, and MMHP NPs for the next 1 d. After coculturing, the cell medium was replaced by the methyl thiazolyl tetrazolium solution (2.5 mg ml^−1^) for the next 4 h of incubation. Then, the methyl thiazolyl tetrazolium solution was extracted and the dimethyl sulfoxide solution was added to the cells for detection with a microplate reader at OD of 490 nm.

### Molecular docking of NPs with NP protein

First, the crystal structure of the complex of nucleoprotein of H1N1 virus, protein ID 2IQH, was downloaded from the Protein Data Bank database and optimized. Then, hydrogen atoms are added, receptors and ligands are separated, and redundant structures are removed. Molecular docking calculations are performed using the Autodock Vina software. After importing the receptor and ligand files, the ligand is placed in the receptor pocket, the ligand conformation is searched/adjusted to obtain a possibility-binding conformation, a box is set up to wrap the pocket, and the ligand molecules are molecularly docked inside the box (searching for conformation and scoring evaluation). We placed the ligand in the receptor pocket and searched the ligand conformation to obtain a possible binding conformation. After that, the structure of HP and NP was gotten and the hydrogen bond was analyzed.

### Quantitative reverse transcription polymerase chain reaction and enzyme-linked immunosorbent assay (ELISA) of NP

After the A549 cells were cocultured with MMHP NPs and H1N1 virus, the cells were extracted and the expressions level was detected with a Bio-Rad instrument. The RNA concentrations were detected using the SYBR Green method. Specifically, the supernatant of the coculture medium of MMHP and A549 cells was first removed, and then the cells were washed twice with PBS using the Total RNA Kit (OMEGA) and tested using the PrimeScript RT Master Mix. So as to the NP protein concentrations, after the A549 cells were cocultured with MM or MMHP NPs and the H1N1 virus, the nucleoprotein was detected using the ELISA method. The ELISA kit is first removed and is left at room temperature for 1 h until its temperature rises to room temperature and remains stable, after which the standard solution is added to the standard dilution, and the process is repeated 5 times and the standard curve is plotted. The standards were then added to the different well plates separately. Subsequently, the detection reagents were added to the standard solution and the samples, and the absorbance of all wells was measured faithfully in a microplate reader (at 450 nm) to calculate the relative protein content.

### Inflammatory factor evaluation in vivo

The mice were divided into 4 groups, including the untreated group (set as the control group), *S. aureus* with 10^7^ CFU ml^−1^ of bacteria and H1N1 virus-treated mice group, MM NP-treated coinfected mice group, and MMHP NP-treated coinfected mice group. The total number of mice was 48, and the in-group replicates were 3. First, the lung of mice was infected with *S. aureus* and the H1N1 virus through the way to drip the nose. After 1 and 2 d of treatment with these NPs, the mice were sacrificed. Then, the lung tissues were extracted and stained with IL-6 and TNF-α. The sections of MMHP samples were put into xylene I for 20 min, xylene II for 20 min, and 75% alcohol for 5 min. Tissue sections of MMHP samples were placed in a microwave oven including a citric acid antigen repair buffer (pH = 6.0) or EDTA antigen repair buffer (pH = 9.0) for antigen repair. After natural cooling, the slides of MMHP samples were placed in PBS (pH = 7.4) and washed. Primary antibodies were added dropwise to the sections, and the sections were incubated flat at 4 °C overnight. Then, the sections were slightly shaken, dried, covered with secondary antibody (horseradish peroxidase-labeled) of the corresponding species in the circle, and incubated for 50 min at room temperature.

### Flow cytometry in vivo in mice lung tissues

After the treatment with MMHP NPs, the serum was collected and analyzed using a whole blood analyzer. The total number of mice was 48, and the in-group replicates were 3. Meanwhile, the serum was stained with CD3/CD4/CD8 and CD206/F4-80 for the next flow cytometry. Each sample was resuspended at 100 μl of PBS, and 1 μl of CD11C antibody was added and incubated at 4 °C for 30 min. Then, the cells of the MMHP samples were washed twice in PBS. Five hundred microliters of 0.5% Triton-X was then added to each tube and permeabilized at room temperature for 10 min. The supernatant of MMHP samples was removed by centrifugation and washed once in PBS. After permeabilization, the MMHP samples were stained with the CD206 antibody. After incubation with the CD206 antibody, the supernatant of MMHP samples was carefully removed after the last wash and resuspended in PBS.

### Virus and bacteria mixed infection model therapy in mice

All mice were purchased from Tianjin Yi Sheng Yuan Biotechnology Co. Ltd., and the animal experiments were conducted in accordance with animal ethics. The total number of mice was 48, and the in-group replicates were 3. The mice (6 weeks) were divided into 4 groups, including the untreated group (set as the control group), *S. aureus* with 10^7^ CFU ml^−1^ of bacteria and H1N1 virus-treated mice group, MM NP-treated coinfected mice group, and MMHP NP-treated coinfected mice group. After the treatment with these NPs, the lung and trachea were collected and plotted. The H&E staining of lung and trachea tissues was carried out for inflammation analysis.

### Intestinal microbiological detection

All mice were purchased from Tianjin Yi Sheng Yuan Biotechnology Co. Ltd., and the animal experiments were conducted in accordance with animal ethics. The total number of mice was 48, and the in-group replicates were 3. The mice (6 weeks) were divided into 2 groups including the PBS and MMHP groups. They were first infected with *S. aureus* (50 μl; injection in situ) and treated with PBS or MMHP NPs (200 μl, 200 μg ml^−1^) the next day. After the mice were sacrificed on day 3, the colons were extracted and collected for taking photos.

### Statistical analysis

All the quantitative data were analyzed by one-way analysis of variance (ANOVA), and the data were presented as the mean with standard deviation.

## Data Availability

The authors declare that the data from this study are available within the paper and the Supplementary Materials, as well as from the authors upon request.

## Supplementary Materials

Supplementary Materials Fig. S1. (A) The TEM image of MM. The scale bar was 20 nm. (B) The relative release of HP under different pH conditions. (C) The release of HP as the culture time gradually increased from 1 to 3 d. (D) The antioxidant activity of MMHP after different times. Fig. S2. The antibacterial effects of PBS, HP, and MM toward *S. aureus* and *E. coli*. Fig. S3. The scanning electron microscope image of *S. aureus*, *E. coli*, *A. ba*, *P. ae*, *S. ty*, and *A. ve* after antibacterial process. Fig. S4. (A) The TEM image of *S. aureus* treated with PBS. (B) The cell uptake efficiency of MMHP NPs. Fig. S5. The H&E staining of the heart, lung, liver, spleen, and kidney treated by PBS and MMNP NPs. Fig. S6. The blood routine examination of PBS, MM, NP, and MMHP NPs at day 3. Fig. S7. The H&E staining of the lung and trachea treated by PBS and MMHP NPs. Table S1. The affinity and RMSD between ATP and HP. Table S2. The affinity and RMSD between HP and different ions. Table S3. The affinity and RMSD between HP and NP protein of 4 kinds of binding mode. Table S4. The microbial alpha diversity of mice gut microbiota treated by PBS and MMHP NPs.
