# Supplementary material for: Inflammation and Microbiota Regulation Potentiate Pneumonia Therapy by Biomimetic Bacteria and Macrophage Membrane Nanosystem
Source: Research (Wash D C). 2023 Mar 27;6:0096. doi: 10.34133/research.0096 (PMC10042321; doi:10.34133/research.0096)
Supplement: Supplementary Materials — Fig. S1. (A) The TEM image of MM. The scale bar was 20 nm. (B) The relative release of HP under different pH conditions. (C) The release of HP as the culture time gradually increased from 1 to 3 d. (D) The antioxidant activity of MMHP after different times. Fig. S2. The antibacterial effects of PBS, HP, and MM toward S. aureus and E. coli. Fig. S3. The scanning electron microscope image of S. aureus, E. coli, A. ba, P. ae, S. ty, and A. ve after antibacterial process. Fig. S4. (A) The TEM image of S. aureus treated with PBS. (B) The cell uptake efficiency of MMHP NPs. Fig. S5. The H&E staining of the heart, lung, liver, spleen, and kidney treated by PBS and MMNP NPs. Fig. S6. The blood routine examination of PBS, MM, NP, and MMHP NPs at day 3. Fig. S7. The H&E staining of the lung and trachea treated by PBS and MMHP NPs. Table S1. The affinity and RMSD between ATP and HP. Table S2. The affinity and RMSD between HP and different ions. Table S3. The affinity and RMSD between HP and NP protein of 4 kinds of binding mode. Table S4. The microbial alpha diversity of mice gut microbiota treated by PBS and MMHP NPs. [file research.0096.f1.docx]

**Supplemental Material**

Inflammation and Microbiota Regulation Potentiates Pneumonia Therapy by Biomimetic Bacteria and Macrophage Membrane Nanosystem

Yuan Li^1,2,3^, Xiangmei Liu^3,4^, Zhenduo Cui^2^, Yufeng Zheng^1^, Hui Jiang^2^, Yu Zhang^5^, Zhaoyang Li^2^, Shengli Zhu^2^, Paul K Chu^6^, Shuilin Wu^1,2,3^*

*Corresponding author. Email: slwu@pku.edu.cn

**This file includes:**

Text

The materials included Supplements Figs. 1-7: The structural characterization of MM and MMHP, antibacterial properties; Table 1: The affinity, RMSD between ATP and HP; Table 2: The affinity, RMSD between HP and different ions. Table 3: The affinity, RMSD between HP and NP protein of four kinds of binding mode. Table 4: The microbial alpha diversity of mice gut microbiota treated by PBS and MMHP NPs.

**Figure S1.** The TEM image of MM. The scale bar was 20 nm. b, the relative release of HP under different pH conditions; c, the release of hypericin as the culture time gradually increased from 1 day to 3 days, d, the antioxidant activity of MMHP after different time.

| Pose | Affinity (kcal mol^-1^) | RMSD_ub (Å) | RMSD_ub (Å) |
| --- | --- | --- | --- |
| 1 | -2.2 | 0 | 0 |
| 2 | -2.2 | 7.572 | 4.639 |
| 3 | -2.1 | 11.526 | 9.836 |
| 4 | -2 | 6.501 | 3.289 |

**Table S1.** The affinity, RMSD between ATP and HP.


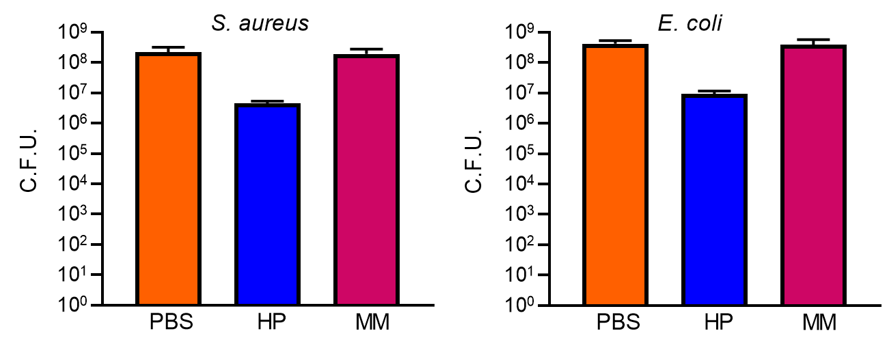


**Figure S2.** The antibacterial effects of PBS, HP, and MM towards *S. aureus* and *E. coli*.

**
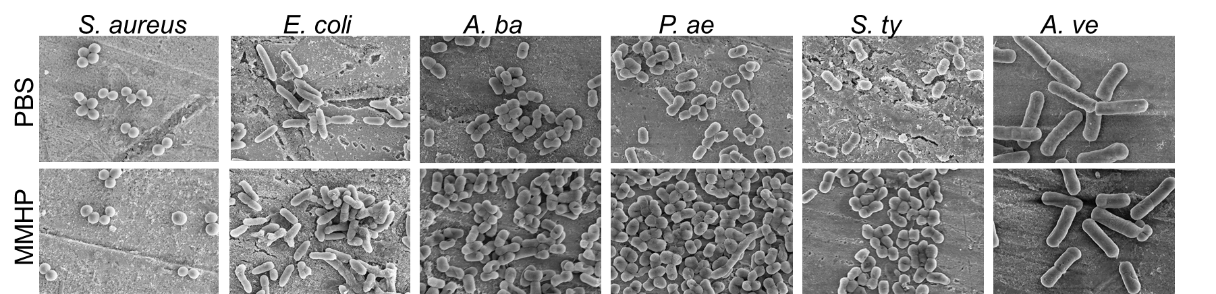
**

**Figure S3.** The SEM image of *S. aureus*, *E. coli*, *A. ba*, *P. ae*, *S. ty,* and *A. ve* after antibacterial process.

| Pose | Affinity (kcal mol^-1^) | RMSD_ub (Å) | RMSD_lb (Å) |
| --- | --- | --- | --- |
| Mn^2+^ | -0.34 | 3.745 | 3.745 |
| Cu^2+^ | -0.56 | 11.528 | 11.528 |
| Mg^2+^ | -0.6 | 4.751 | 4.751 |
| Fe^3+^ | -0.22 | 11.196 | 11.196 |

**Table S2.** The affinity, RMSD between HP and different ions.

b

a

**

**
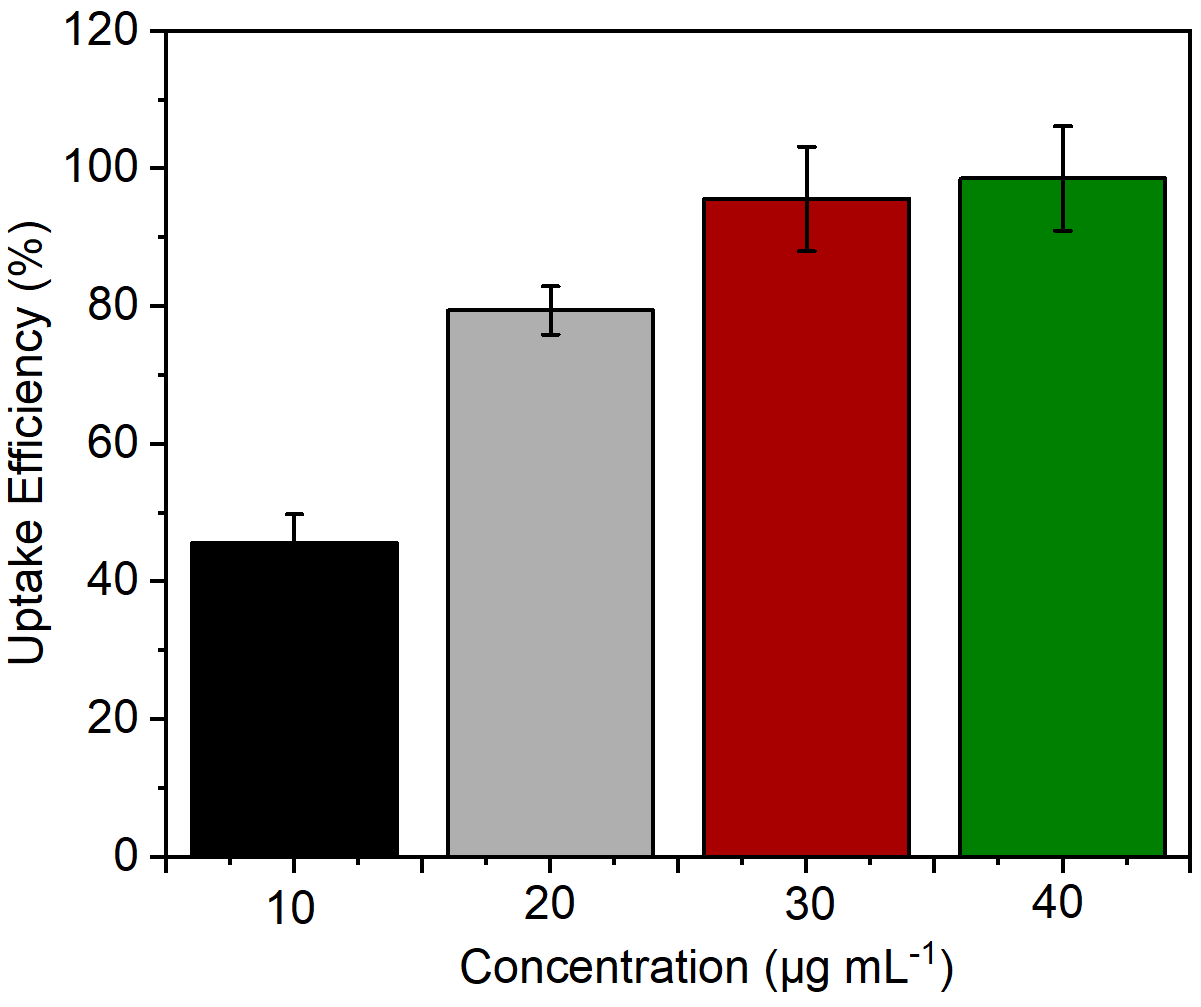

**Figure S4.** a, the TEM image of *S. aureus* treated with PBS; b, the cell uptake efficiency of MMHP NPs.

| Pose | Affinity (kcal mol^-1^) | RMSD_ub (Å) | RMSD_lb (Å) |
| --- | --- | --- | --- |
| 1 | -8.8 | 5.708 | 0.133 |
| 2^+^ | -8.7 | 6.369 | 2.739 |
| 3 | -8.6 | 5.957 | 1.389 |
| 4 | -8.6 | 4.026 | 1.434 |

**Table S3.** The affinity, RMSD between HP and NP protein of four kinds of binding mode.


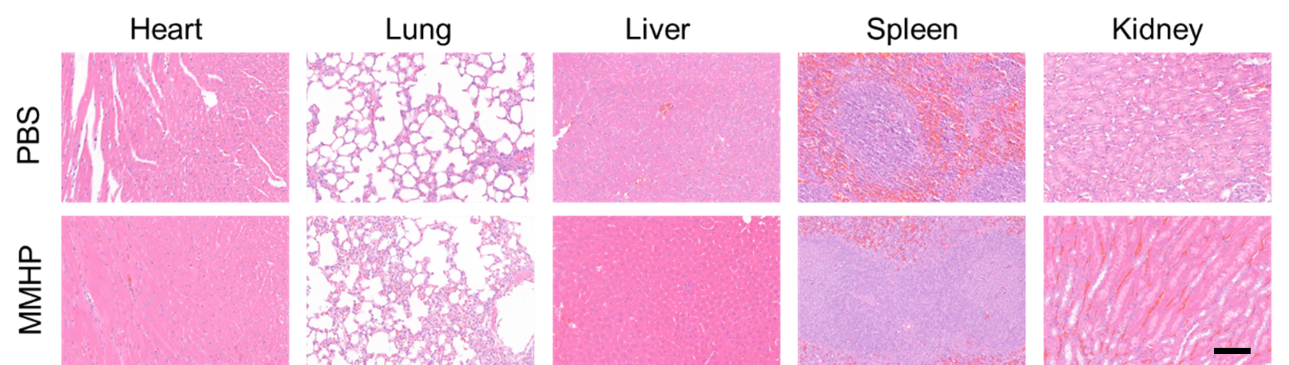


**Figure S5.** The H&E staining of heart, lung, liver, spleen, and kidney treated by PBS and MMNP NPs.

**
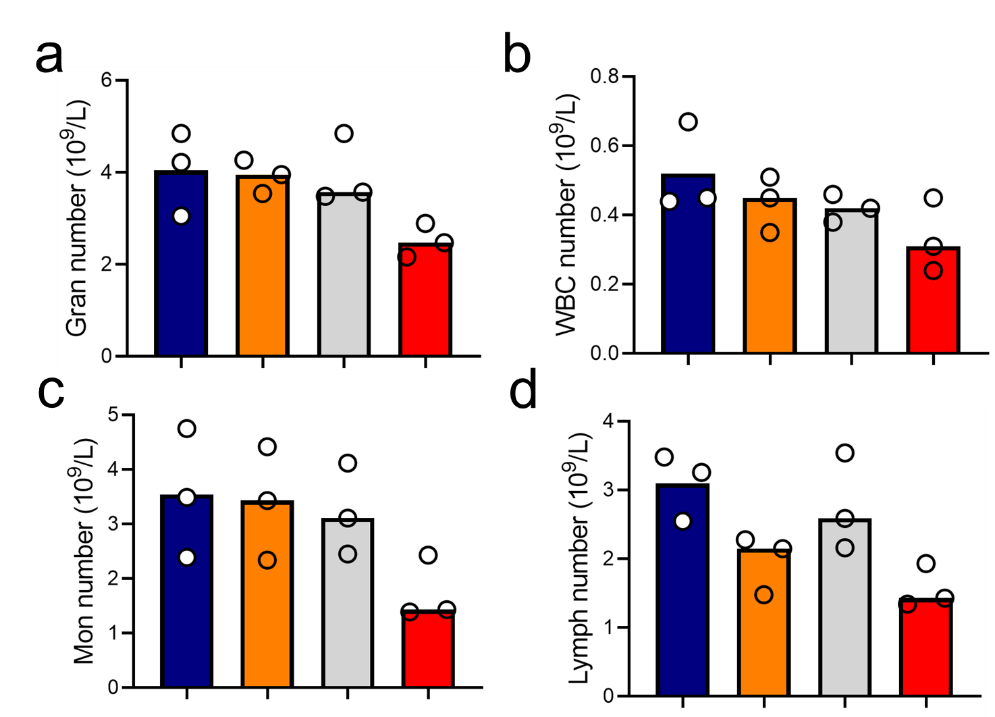
**

**Figure S6.** The blood routine examination of PBS, MM, NP, and MMHP NPs at day 3.


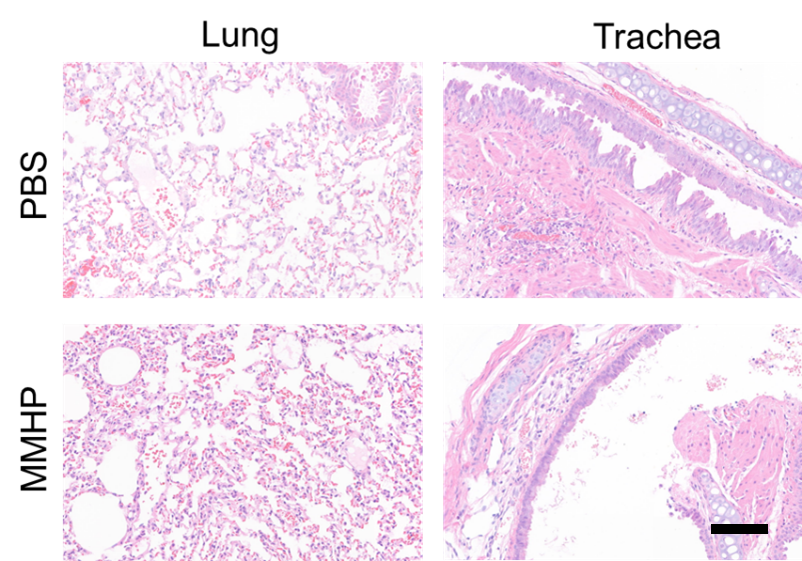


**Figure S7.** The H&E staining of lung and trachea treated by PBS and MMHP NPs.


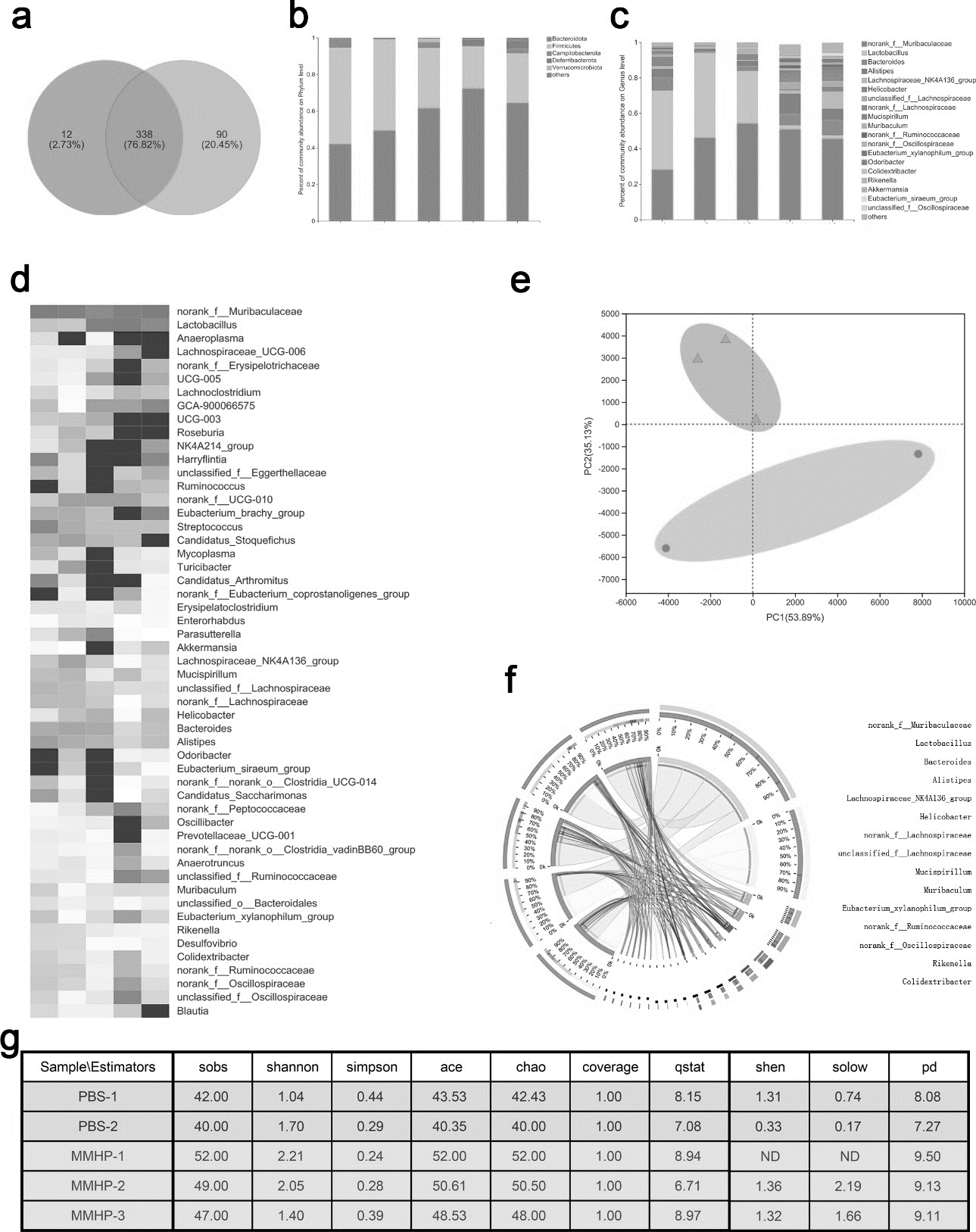


**Table S4.** The microbial alpha diversity of mice gut microbiota treated by PBS and MMHP NPs.
